# Neither a Novel Tau Proteinopathy nor an Expansion of a Phenotype: Reappraising Clinicopathology-Based Nosology

**DOI:** 10.3390/ijms22147292

**Published:** 2021-07-07

**Authors:** Luca Marsili, Jennifer Sharma, Alberto J. Espay, Alice Migazzi, Elhusseini Abdelghany, Emily J. Hill, Kevin R. Duque, Matthew C. Hagen, Christopher D. Stephen, Gabor G. Kovacs, Anthony E. Lang, Marios Hadjivassiliou, Manuela Basso, Marcelo A. Kauffman, Andrea Sturchio

**Affiliations:** 1 Gardner Family Center for Parkinson’s Disease and Movement Disorders, Department of Neurology and Rehabilitation Medicine, University of Cincinnati, Cincinnati, OH 45219, USA; sharmajr@ucmail.uc.edu (J.S.); espayaj@ucmail.uc.edu (A.J.E.); elhusshe@ucmail.uc.edu (E.A.); hill2e9@ucmail.uc.edu (E.J.H.); duqueykr@ucmail.uc.edu (K.R.D.); sturchaa@ucmail.uc.edu (A.S.); 2 Laboratory of Transcriptional Neurobiology, Department of Cellular, Computational and Integrative Biology—CIBIO, University of Trento, 38123 Trento, Italy; alice.migazzi@unitn.it (A.M.); manuela.basso@unitn.it (M.B.); 3 Department of Pathology, University of Cincinnati, Cincinnati, OH 45219, USA; hagenmw@ucmail.uc.edu; 4 Ataxia Center, Department of Neurology, Massachusetts General Hospital, Harvard Medical School, Boston, MA 02114, USA; cstephen@mgh.harvard.edu; 5 Tanz Centre for Research in Neurodegenerative Disease (CRND), Department of Laboratory Medicine and Pathobiology, University of Toronto, 60 Leonard Ave, Krembil Discovery Tower, Toronto, ON M5T 0S8, Canada; gabor.kovacs@uhnresearch.ca; 6 Laboratory Medicine Program and Krembil Brain Institute, University Health Network, Toronto, ON M5T 1M8, Canada; 7 Edmond J. Safra Program in Parkinson’s Disease, Rossy Progressive Supranuclear Palsy Program and the Morton and Gloria Shulman Movement Disorders Clinic, Toronto Western Hospital, University of Toronto, Toronto, ON M5T 2S8, Canada; Anthony.Lang@uhnresearch.ca; 8 Academic Department of Neurosciences, Royal Hallamshire Hospital, University of Sheffield, Sheffield S10 2JF, UK; m.hadjivassiliou@sheffield.ac.uk; 9 Consultorio y Laboratorio de Neurogenética, Centro Universitario de Neurología José María Ramos Mejía, Buenos Aires C1221ADC, Argentina; marcelokauffman@gmail.com

**Keywords:** cerebellar ataxia, neurogenetics, movement disorders, postmortem

## Abstract

The gold standard for classification of neurodegenerative diseases is postmortem histopathology; however, the diagnostic odyssey of this case challenges such a clinicopathologic model. We evaluated a 60-year-old woman with a 7-year history of a progressive dystonia–ataxia syndrome with supranuclear gaze palsy, suspected to represent Niemann–Pick disease Type C. Postmortem evaluation unexpectedly demonstrated neurodegeneration with 4-repeat tau deposition in a distribution diagnostic of progressive supranuclear palsy (PSP). Whole-exome sequencing revealed a new heterozygous variant in *TGM6*, associated with spinocerebellar ataxia type 35 (SCA35). This novel *TGM6* variant reduced transglutaminase activity in vitro, suggesting it was pathogenic. This case could be interpreted as expanding: (1) the PSP phenotype to include a spinocerebellar variant; (2) SCA35 as a tau proteinopathy; or (3) *TGM6* as a novel genetic variant underlying a SCA35 phenotype with PSP pathology. None of these interpretations seem adequate. We instead hypothesize that impairment in the crosslinking of tau by the *TGM6*-encoded transglutaminase enzyme may compromise tau functionally and structurally, leading to its aggregation in a pattern currently classified as PSP. The lessons from this case study encourage a reassessment of our clinicopathology-based nosology.

## 1. Introduction

The gold standard for classification of neurodegenerative diseases is postmortem histopathology. Tau is a microtubule-associated protein, which, when hyperphosphorylated, accumulates in unique patterns dependent on selective regional vulnerability [1,2,3]. Tau accumulation is associated with neurodegenerative diseases currently labeled as tauopathies. Phenotypes associated with abnormal tau accumulation include, but are not limited to, progressive supranuclear palsy (PSP), corticobasal degeneration, Pick’s disease, argyrophilic grain disease, and several less common conditions [4]. The range of “tau proteinopathy” disorders, defined by the accumulation of filamentous tau at autopsy, is continuously growing and has begun to include clinically unrelated disorders [5].

Here, we describe the intellectual odyssey of a case with initially discrepant clinical, genetic, and pathologic information in whom the confirmation of pathogenicity of a novel genetic variant in *TGM6*, a genotype without prior reported neuropathology, prompted reinterpretation of the data and a reappraisal of the clinicopathology model on which the nosology of tau proteinopathies is based.

## 2. Case Report

A 59-year-old right-handed Caucasian woman presented to our center after a five-year history of jerks and stiffness in the left limbs, frequent falls, dysphagia, and mild emotional lability. She had first noticed loss of fine motor skills in her left hand while typing. This progressed over a year to include “spasms” when attempting to utilize that hand. Within two years, she exhibited jerky movements and intermittent painless posturing of her left foot, lasting from seconds to minutes. Four years after symptom onset, she developed progressive dysphagia, falls, worsened rigidity, and a left leg that was “not following instructions.” She manifested compulsive behaviors and inappropriate crying and laughing. She denied dietary or bowel habit changes, anosmia, dream-enactment behaviors, or any other sleep-related symptoms. She occasionally repeated questions but had no cognitive complaints. While many aspects of her family history could not be confirmed, her deceased maternal grandmother had apparent dysphonia and tremor. Her parents and siblings had no neurological diseases.

Neurological examination revealed a complex hyperkinetic syndrome consistent with dysarthria, orofacial dystonia, eye movement abnormalities (oculomotor apraxia, slowed horizontal saccades, severe vertical gaze palsy, overcome with oculocephalic maneuvers), and multidirectional, jerky, low-frequency head movements that were variably interpreted as tremor or stereotypies against a background of generalized dystonia that was greater in the left limbs and included a right hand tremor with cerebellar and dystonic features (Supplementary Video S1). The Montreal Cognitive Assessment revealed predominantly frontal cognitive impairment (21/30) with deficits in visuospatial acuity (2/5), serial subtractions (0/3), verbal fluency (0/1), and delayed recall (3/5) tasks.

**Figure 1:**
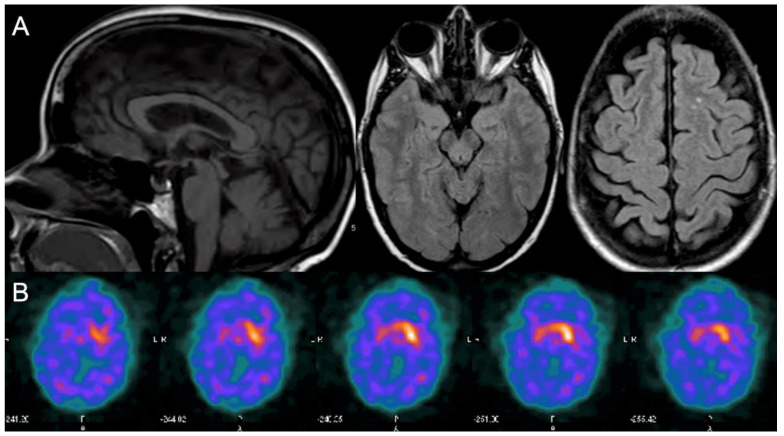
Brain magnetic resonance imaging (MRI) and single-photon emission computed tomography (DaTscan) obtained four years after symptom onset. (**A**) Brain MRI Left panel: mid-sagittal T1-weighted sequence with mild vermal cerebellar atrophy and midbrain atrophy (midbrain anteroposterior diameter, 8.6 mm; midbrain-to-pons ratio 0.54) [6]; central panel: axial T2 FLAIR sequence showing mild tegmental midbrain atrophy; right panel: axial T2 FLAIR sequence showing two small white matter hyperintensities. (**B**) DaTscan showed asymmetric (right > left), abnormally decreased uptake in the bilateral putamen and right caudate.

Laboratory investigations revealed normal serum copper, ceruloplasmin, heavy metals, vitamin E, comprehensive metabolic profile (including liver function tests), mildly high ferritin with normal iron, and no acanthocytes. She had no antibodies against phospholipids, double-stranded DNA, glutamic acid decarboxylase, and gliadin. A serum paraneoplastic antibody panel was negative. Neither copper urine levels nor cerebrospinal fluid analyses were performed. Gene testing for Huntington’s disease was normal. She had a normal electroencephalogram, electromyogram, and nerve conduction studies. Brain magnetic resonance imaging (MRI), obtained four years after symptom onset, revealed mild cerebellar and tegmental midbrain atrophy and scattered white matter hyperintensities (Figure 1A). Cervical spine MRI showed minor spondylotic changes without cord abnormalities. Single-photon emission computed tomography (DaTscan) revealed marked decreased uptake in the bilateral putamina (right > left) and right caudate (Figure 1B). The striatum-binding ratio was 85% below the mean in the right and 68% below in the left.

Based on the clinical features, Niemann–Pick Disease Type-C (NPC) was suspected, but oxysterol levels (<0.02 nmol/mL) and *NPC1* and *NPC2* gene sequencing were normal (skin biopsy not performed). There was relentless progression. By the sixth year of her illness, she had become immobile and lost substantial weight (the last measured weight was below 30 kg) due to dysphagia to both liquids and solids (Supplementary Video S2), with subsequent malnutrition. Trihexyphenidyl and diazepam attenuated the dystonia severity; levodopa, escalated to 600 mg/day, had no effect. She died at the age of 60, 7 years after symptom onset. 

**Figure 2:**
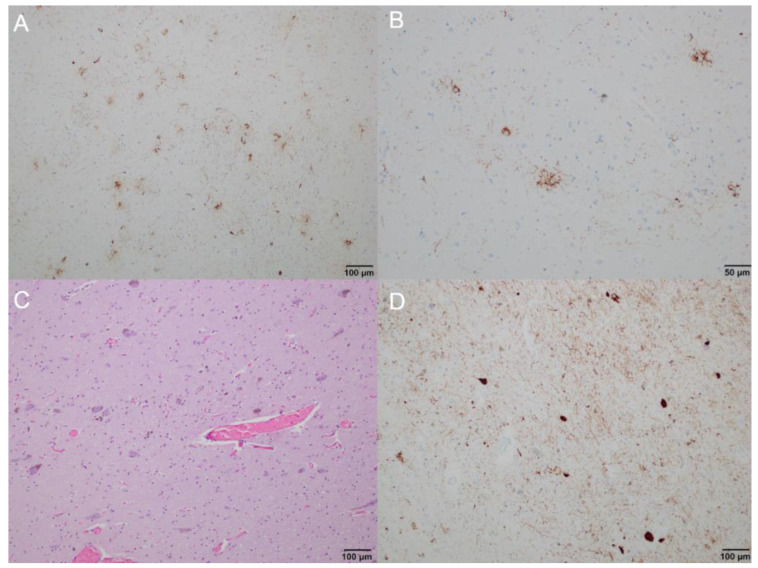
Brain neuropathology, microscopic anatomy: (**A**) immunohistochemistry staining showing tau accumulation in the putamen, predominantly in the form of tufted astrocytes and tau-positive neurons (100× magnification); (**B**) immunohistochemistry staining showing tufted astrocytes in the caudate nucleus (200× magnification); (**C**,**D**) substantia nigra showing neuronal cell loss, tau-positive neurons, and numerous neuropil threads ((**C**) hematoxylin and eosin staining; (**D**) immunohistochemistry staining; 100× magnification).

**Figure 3:**
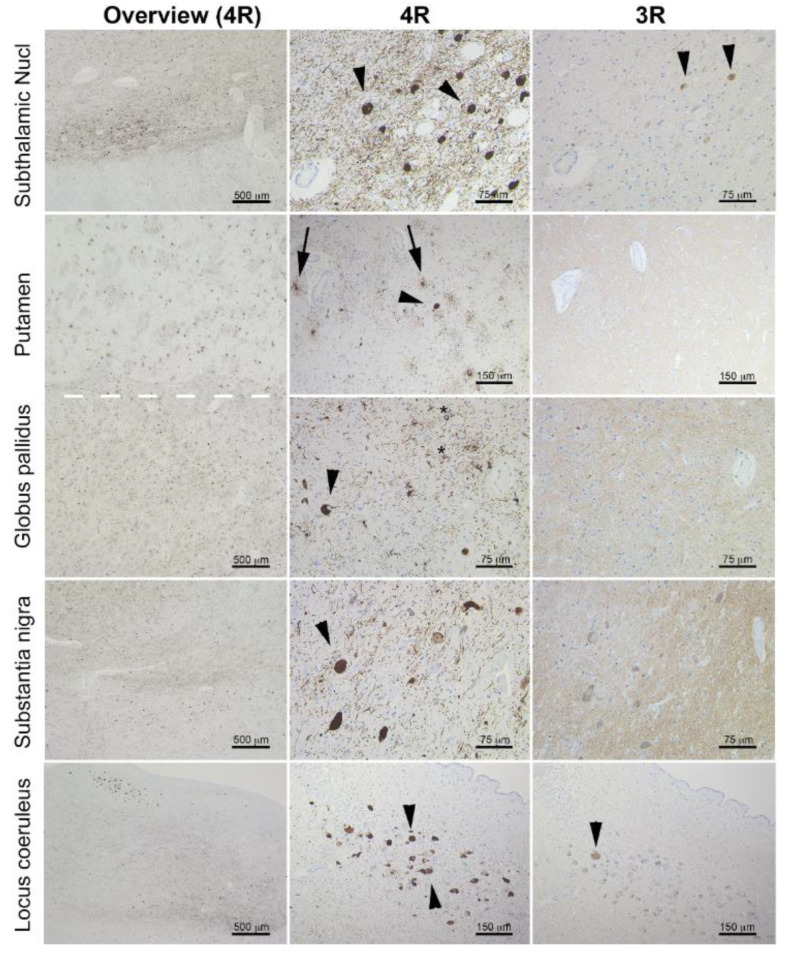
Immunostaining for 4R (left and middle column) and 3R (right column) tau isoforms in the subthalamic nucleus, putamen, globus pallidus, substantia nigra, and locus coeruleus. Note that the pathology is predominated by 4R tau deposition and shows tufted astrocytes (few examples indicated by arrows), globose neurofibrillary tangles (few examples indicated by arrowheads), and coiled bodies (few examples indicated by asterisks). Only a single 3R tau immunoreactive neurofibrillary tangle is seen in the locus coeruleus (arrowhead), and two are seen weakly stained in the subthalamic nucleus (arrowheads).

**Postmortem neuropathology.** On gross examination, the brain was mildly atrophic (1050 g); there was marked hypopigmentation in the substantia nigra and mild atrophy of the anterior cerebellar vermis and brainstem. Neuropathology examination was performed using formalin-fixed paraffin-embedded tissue blocks of several cortical, subcortical, and brainstem regions, as well as the cerebellum. The following monoclonal antibodies were used for immunohistochemistry: anti-tau (clone AT8), anti-4 repeat(R)tau (RD4), anti-3R tau (RD3), anti-phospho-TDP-43 (pS409/410; GTX82580), anti-α-synuclein (5G4; SPM451), and anti-Aβ (6F/3D; 2C8). Immunostaining for AT8 revealed neurofibrillary tangles (seen also on hematoxylin-eosin staining), fine granular cytoplasmic immunoreactivity in neuronal cytoplasm, neuropil threads, tufted astrocytes, oligodendrocytic-coiled bodies, and threads in the white matter (Figure 2A–D). The bulk of neuronal and oligodendrocytic tau pathology was in subcortical areas, including the subthalamic nucleus, midbrain, pons, medulla oblongata, tegmentum, and substantia nigra. The dentate nucleus and the anterior horn of the spinal cord were also affected. Astrocytic tau pathology predominated in the striatum and cortical regions, particularly in the frontal cortex, but was also seen in the occipital cortex. The extensive immunohistochemical staining for beta amyloid on the left superior and middle temporal gyri, left middle frontal gyrus, left inferior parietal lobule, and right cerebellum did not reveal any amyloid angiopathy, diffuse deposits, or senile/neuritic plaques. Immunostaining for 4R tau isoform was positive, while immunostaining for 3R tau isoform was not (Figure 3). TDP-43, beta-amyloid, and alpha-synuclein staining were negative. In summary, the neuropathological features were diagnostic of the 4R tau proteinopathy, PSP.

**Genetic evaluation.** Whole-exome sequencing revealed a heterozygous variant of unknown significance (NM_198994 c.616A > C, p.T206P) in *TGM6*, associated with spinocerebellar ataxia type 35 (SCA35) [2] but previously unreported.

**Figure 4:**
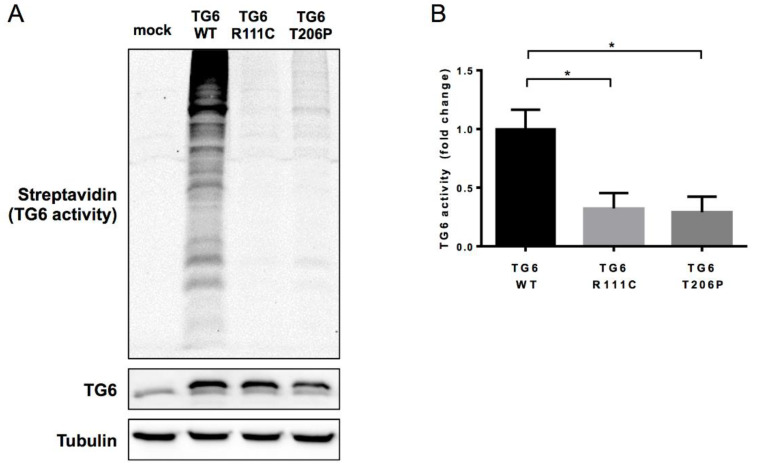
In vitro assay showing TG6 transamidase activity: (**A**) Western blotting analysis showed significantly compromised transamidase activity of the *TGM6* T206P variant compared to wild-type TG6 protein. (**B**) Quantification of TG6 enzymatic activity from experiment shown in panel A. Graph, mean ± SEM, * *p* <0.05, one-way ANOVA with Tukey’s post hoc test. Y-Axis represents the fold change.

**Functional evaluation of the T206P-*TGM6* variant.** The *TGM6* protein transglutaminase 6 (TG6) catalyzes transamidation (crosslinking) within and between proteins. Pathogenic *TGM6* variants have shown a loss in transamidase activity and increased neuronal toxicity in vitro and in vivo [7,8]. Therefore, to assess for pathogenicity, we tested whether the T206P variant affected the TG6 enzymatic activity involved in the transamidation of glutamine. We cloned the T206P variant in a mammalian expression vector, transfected HEK293T cells, and tested TG6 transamidase activity in vitro [8]. T206P significantly reduced TG6 activity when compared to wild-type TG6 (Figure 4), and the reduction in transamidase activity was similar to that observed in the R111C variant previously identified in SCA35 patients and reported as toxic in primary neurons and *drosophila*. The loss in enzymatic function supported the pathogenic role of T206P in our patient.

## 3. Discussion

The clinical picture of a markedly asymmetric, progressive ataxia–dystonia syndrome with oculomotor apraxia and vertical supranuclear gaze palsy resembled NPC but was instead associated with a new pathogenic variant (T206P) in *TGM6*, previously reported as the genetic etiology of SCA35, and with an unanticipated pathology consistent with PSP. While the clinical features were broadly compatible with the syndrome of spinocerebellar ataxia, SCA35 has never been reported to be associated with supranuclear gaze palsy (as per a systematic review of 66 cases reported; Supplementary Table S1 [9,10,11,12,13,14,15] and Figure S1), nor with an underlying 4R tau proteinopathy compatible with PSP pathology. That said, in the background of severe DaT deficit on imaging and vertical supranuclear gaze palsy on examination, PSP was clinically excluded by the hyperkinetic rather than the parkinsonian phenotype, as well as the presence of appendicular and axial ataxia, which are exclusionary [16]. While a cerebellar variant of PSP has been reported [17], these patients, mostly from Asia, have not manifested dystonia or other hyperkinesias, nor the marked dopamine transporter loss exhibited by our patient. Moreover, the distribution of tau pathology did not show a cerebellum-predominant pattern; instead, it met staging five (of six) for PSP–Richardson syndrome [18]. We recognize that the clinical perspective on what represents a “tau proteinopathy”, with the accumulation of filamentous tau, continues to evolve, given the growing range of clinically unrelated disorders with documented tau accumulation at autopsy [5]. 

TG6 mutations have been reported to decrease its transamidase activity and increase susceptibility to apoptosis in neuronal [8] and non-neuronal cells [19]. *TGM6* codes for TG6, a calcium-dependent enzyme expressed in the central nervous system, particularly in the olfactory lobe, cerebral cortex, cerebellum, and brainstem [20]. TG6 activity is responsible for creating stable isopeptide bonds, also known as transglutaminase-catalyzed protein crosslinking [20,21]. Transglutaminases can also polyaminate proteins, namely they add primary amines to lysines and glutamines [1]. Polyamination of tubulin has been shown to be essential for the stabilization of axon microtubules [1,2]. Alterations in tubulin polyamination by transglutaminases could favor the formation of neurofibrillary tangles, a feature of several tau-related disorders [3].

Abnormal non-specific transglutaminase activity has been previously identified in Alzheimer’s disease, Huntington’s disease, and PSP [2,3,22]. Under physiologic conditions, transglutaminase activity and the corresponding stability of microtubules are essential for neural structure and function, as well as early neurite development [13]. The inhibition of transglutaminase activity reduces microtubules’ stability in culture [1]. Although transglutaminase activity and levels of microtubules are relatively low in immature cells, they concurrently increase with differentiation, facilitating extension and stabilization of neurites [1,2,3]. These changes in cytoskeletal dynamics and stability in neurodegenerative processes, while incompletely understood, result in polymerization of the protein with subsequent loss of their function [1,2,3]. The observable result is the accumulated protein, the anchor used to classify and approach neurodegenerative disorders; the unobservable event is the loss of the functional, soluble precursors of the same protein (as they turn into tangles), which is invisible at autopsy. 

Our case highlights a nosology challenge: whether to describe it as expanding the phenotypic spectrum associated with PSP pathology or with *TGM6*-SCA35. On one hand, our observation of an NPC-like phenotype adds to the variability of clinical presentations associated with PSP-type tau pathology (interestingly, 8.5% PSP patients harbor heterozygous variants in *NPC1* or *NPC2*) [23]. On the other hand, since we detected a genetic variant associated with SCA35, and this gene is related to an enzyme associated with functional impairment of tau crosslinking, we theorize that aberrant TG6 might contribute to the initiation of tau pathology in a distribution currently classified as PSP. 

The present case has several limitations. Unfortunately, we were unable to study the deceased maternal grandmother or her healthy siblings to evaluate for *TGM6* carrier status. We also acknowledge that a single case study cannot address the wider question of whether the pathogenetic mechanisms of tau proteinopathies are associated with normal tau depletion (loss-of-function model) [24] or abnormal tau accumulation (gain-of-function model), and the extent to which either or both of these contribute to the underlying clinical phenotype [25]. Further in vivo studies on mouse models will be needed to clarify the interaction between *TGM6* and tau pathology. Tentatively, our observations lend support to the notion that tau pathology, even in a pattern currently classified as PSP, may be the result of several upstream disrupted pathogenic abnormalities (TG6-associated impaired tau crosslinking among them) but not *the* pathogenic abnormality. The clinico-genetic-pathology discrepancies highlighted by this case may serve to inform a future reassessment of the clinicopathology-based nosology of neurodegenerative disorders. 

## Data Availability

Not applicable.

## Supplementary Materials

The following are available online at https://drive.google.com/drive/folders/1Db_4b5sBaL-DwtCYdkcwHWatiFYV5hFS, Supplementary Video S1. Neurologic examination of the patient at the first visit, five years after symptom onset. Supplementary Video S2. Second neurologic examination, six month after the first video. The patient is increasingly immobile, exhibiting marked weight loss due to dysphagia. The left arm has become nearly fixed. Supplementary Table S1. TGM6 variants reported in literature and related clinical features. Supplementary Figure S1. Lollipop graph depicting the TGM6 gene with the known variants located on their specific positions.
